# Principles of Motor Learning to Support Neuroplasticity After ACL Injury: Implications for Optimizing Performance and Reducing Risk of Second ACL Injury

**DOI:** 10.1007/s40279-019-01058-0

**Published:** 2019-02-05

**Authors:** Alli Gokeler, Dorothee Neuhaus, Anne Benjaminse, Dustin R. Grooms, Jochen Baumeister

**Affiliations:** 1 Exercise Science & Neuroscience Unit, Department Exercise and Health, Faculty of Science, Paderborn University, Paderborn, Germany; 2 Luxembourg Institute of Research in Orthopedics, Sports Medicine and Science (LIROMS), Luxembourg, Luxembourg; 3 Center for Human Movement Sciences, University of Groningen, University Medical Center Groningen, Groningen, The Netherlands; 4 School of Sport Studies, Hanze University Groningen, Groningen, The Netherlands; 5 Division of Athletic Training, School of Applied Health Sciences and Wellness, College of Health Sciences and Professions, Ohio University, Athens, OH USA; 6 Ohio Musculoskeletal and Neurological Institute, Ohio University, Athens, OH USA; 7 Exercise Neuroscience and Health Lab, Institute of Health, Nutrition and Sport Sciences, University of Flensburg, Flensburg, Germany; 8 Division of Physiotherapy/Central Analytical Facilities (CAF) 3D Human Biomechanics Unit, Faculty of Medicine and Health, Stellenbosch University, Stellenbosch, South Africa

## Abstract

Athletes who wish to resume high-level activities after an injury to the anterior cruciate ligament (ACL) are often advised to undergo surgical reconstruction. Nevertheless, ACL reconstruction (ACLR) does not equate to normal function of the knee or reduced risk of subsequent injuries. In fact, recent evidence has shown that only around half of post-ACLR patients can expect to return to competitive level of sports. A rising concern is the high rate of second ACL injuries, particularly in young athletes, with up to 20% of those returning to sport in the first year from surgery experiencing a second ACL rupture. Aside from the increased risk of second injury, patients after ACLR have an increased risk of developing early onset of osteoarthritis. Given the recent findings, it is imperative that rehabilitation after ACLR is scrutinized so the second injury preventative strategies can be optimized. Unfortunately, current ACLR rehabilitation programs may not be optimally effective in addressing deficits related to the initial injury and the subsequent surgical intervention. Motor learning to (re-)acquire motor skills and neuroplastic capacities are not sufficiently incorporated during traditional rehabilitation, attesting to the high re-injury rates. The purpose of this article is to present novel clinically integrated motor learning principles to support neuroplasticity that can improve patient functional performance and reduce the risk of second ACL injury. The following key concepts to enhance rehabilitation and prepare the patient for re-integration to sports after an ACL injury that is as safe as possible are presented: (1) external focus of attention, (2) implicit learning, (3) differential learning, (4) self-controlled learning and contextual interference. The novel motor learning principles presented in this manuscript may optimize future rehabilitation programs to reduce second ACL injury risk and early development of osteoarthritis by targeting changes in neural networks.

## Key Points

**Table:** 

Anterior cruciate ligament (ACL) injury has been shown to cause changes in the brain that may not be sufficiently targeted with current rehabilitation approaches.
Using principles of motor learning may have the potential to support neuroplastic processes to reduce second ACL injury risk and the incidence of early osteoarthritis.

## Introduction

Injuries of the anterior cruciate ligament (ACL) are one of the most common and devastating sports injuries. The short-term physical and psychosocial consequences of an ACL injury are significant. Athletes experience limitations in daily life and reduction in sports participation. ACL injuries are also associated with long-term clinical sequelae that include meniscal tears, (osteo-)chondral lesions, and an increased risk of early onset of osteoarthritis [1–3]. Treatment options are either non-surgical or surgical but, irrespective of chosen treatment, usually entail a lengthy rehabilitation [4, 5]. The desire to return to the pre-injury level of sports participation, particularly in sports involving cutting, pivoting, and jumping maneuvers, is a major reason for ACL reconstruction (ACLR) [6]. Studies indicate that after ACLR, on average 81% of athletes returned to any sport, 65% returned to their pre-injury level of sport, and 55% returned to competitive-level sports [7]. Although surgical techniques have continuously improved, asymmetries in motor control during daily and athletic tasks are consistently noted following ACL injury and/or subsequent ACLR [8–12]. The current rehabilitation programs do not effectively target aberrant movement patterns and motor control [13]. Altered movement control has been associated with increased risk for ipsi- or contralateral second injury and the development of early onset of osteoarthritis of the knee joint [14–17]. Changes in kinematics after ACL injury in turn may result in certain areas of cartilage being exposed to altered levels of stress and tensions, whilst other areas may become unloaded. The injury-altered gross lower extremity movement profile results in changes to tibiofemoral contact forces, which may lead to early development of osteoarthritis after ACL injury [14]. Biomechanical and neuromuscular risk factors for second ACL injury include altered hip rotation moments in the uninvolved leg, increased frontal plane knee motion during landing, sagittal plane knee moment asymmetries at initial contact, and deficits in postural stability in the reconstructed leg [17].

For young athletes (< 25 years of age) returning to competitive sports involving jumping and cutting activities, second ACL injury rates of 23% have been reported, especially in the early return-to-sport (RTS) period [18]. Based on the aforementioned continued neuromuscular control deficits, it is apparent that traditional rehabilitation does not restore normal motor function in all patients after ACLR. Components of current rehabilitation programs entail a combination of exercises to increase muscle strength and endurance and improve neuromuscular function [19–22]. Although we acknowledge the importance of addressing these factors, there is a clear need for improvement in light of early development of osteoarthritis and second ACL injury risk. The purpose of this article is to present novel clinically integrated motor learning principles to support neuroplasticity that can improve patient functional performance and reduce the risk of second ACL injury.

## Knowledge from Motor Learning

Motor learning is defined as the process of an individual’s ability to acquire motor skills with a relatively permanent change in performance as a function of practice or experience [23]. The currently most used method to test motor learning is to assess the behavioral resultant outcome [23]. Instructions and supplementary feedback are important influencing factors to support motor learning processes. In almost any training situation where motor skills are to be learned, athletes are given instructions about the correct movement pattern or technique [24]. Instructional language has an influential role on movement performance as well as on motor learning outcomes [25, 26]. To gain expertise and induce a motor learning adaptation, a skill must be rehearsed repeatedly. However, there are many variables to consider when structuring the way practice should proceed, including the amount, the type, and the schedule. The best practice design should not simply promote immediate performance effects but ensure long-term learning by promoting retention and transfer of skills. In addition, task-specific or task-oriented practice should be used that is meaningful to the patient. Further, it is important to ensure the task practiced is challenging and motivating for the patient.

The following key concepts may enhance rehabilitation by targeting movement asymmetries and prepare the patient for re-integration to sports after an ACL injury that is as safe as possible:

1. External focus of attention.
2. Implicit learning.
3. Differential learning.
4. Self-controlled learning and contextual interference.

### External Focus of Attention Improves Motor Learning

In almost any rehabilitation situation where motor skills are to be (re-)learned, patients receive instructions about the deemed correct movement technique. These instructions typically refer to the coordination of the patient’s body movements, including the order, form, and timing of various limb movements.

Instructions that direct the patient’s attention to their own movements induce an internal focus of attention [27]. For example, to increase knee extension during gait, a physical therapist instructs the patient to straighten the knee more during the stance phase. It has been shown that 95% of physical therapists provide instructions with such an internal focus [28].

However, a growing body of evidence shows that this type of attentional focus may not be as effective as previously thought [29]. Interestingly, a simple change in the wording of instructions can have a significant impact on performance and learning. Directing the patient’s attention to the effects of the movements on the environment—an external focus of attention—results in more effective and efficient movements [30]. In this case, to increase knee extension during gait, a physical therapist instructs the patient to pretend to kick a ball during the end of the swing phase. An external focus of attention speeds up the learning process—or shortens the first stages of learning—by facilitating movement automaticity [31]. A focus on the movement effect promotes the utilization of unconscious or automatic processes, whereas an internal focus on one’s own movements results in a more conscious type of control that constrains the motor system and disrupts automatic control processes [31]. Wulf et al. have termed this ‘constrained-action hypothesis’ as the explanation for the attentional focus phenomenon [31]. Support for this view comes from studies showing reduced attentional demands when performers adopt an external as opposed to an internal focus, as well as a higher frequency of low-amplitude movement adjustments, which is seen as an indication of a more automatic, reflex-type mode of control [24]. Taken together, there is a large body of evidence that shows the beneficial effects of using external focus instructions over internal focus instructions [24]. In relation to neuromuscular training exercises specifically targeted at reducing ACL primary and secondary injury risk, it has been clearly established that using instructions with an external focus results in better motor performance than using instructions with an internal focus of attention [32].

#### Neural Correlates of External Focus of Attention

Recent work has begun to shed some light on the neural mechanisms of attentional focus, demonstrating that external focus instructions increase intracortical inhibition, providing neurophysiologic support for the constrained action hypothesis [33]. As cortical activity is influenced by the balance between inhibitory and excitatory circuits, the capability of attentional focus to affect the interactions between excitatory and inhibitory intracortical processes within M1 provide a means to directly influence how the nervous system is generating motor control in real time. The integrated motor, sensory, and insular activity may provide the neurophysiologic mechanism for the typically observed improvements in motor performance related to precise timing, hand–eye coordination, and other tasks that involve coordinated action in response to external stimuli. As ACL injury may alter intracortical facilitation [34] and depressed intracortical inhibition is correlated with decreased quadriceps voluntary activation capability [35], external focus training may provide a means to restore quadriceps muscle activity via increasing intracortical inhibition. Adopting an external focus thus may not only be a means to alter motor behavior for complex and coordinated tasks as typically utilized but can be employed early on and throughout therapy to target an underlying neurophysiologic mechanism (intracortical inhibition), in part contributing to the quadriceps activation failure associated with ACL injury.

#### Clinical Examples

Recent work has also demonstrated the applicability of using an external focus of attention during rehabilitation after ACLR to improve movement skill acquisition that can reduce secondary ACL injury risk [36]. Patients received an instruction with either an internal focus or an external focus before performing a single-leg hop jump. The instructions for the internal focus group were “Jump as far as you can. While you are jumping, I want you to think about extending your knees as rapidly as possible” and for the external focus group “Jump as far as you can. While you are jumping, I want you to think about pushing yourself off as hard as possible from the floor.” During landing, the external focus group had significantly larger knee flexion angles at initial contact, peak knee flexion, total range of motion, and time to peak knee flexion [36].

In addition to jump-landing motor control, postural stability deficits were shown to also be a risk factor for sustaining a second injury after ACLR [17]. As such, postural stability deficits are a potential target for the application of new motor learning principles to reduce the potential for this deficit to remain after return to activity. For example, in patients who had sustained an ankle sprain, an external focus resulted in better postural stability compared with promoting an internal focus [37]. There was an improvement immediately following training and, more importantly, improvement was maintained in a retention test. In the following section, examples are provided of how instructions with an external focus of attention can be employed to improve postural stability. For comparison, instructions with an internal focus of attention are also presented as these are commonly used in daily practice. Clinicians can appreciate that the differences between instructions with an internal and external focus are subtle, but these subtle differences can have large implications on how the nervous system generates motion in a way that can facilitate reduced injury risk motor pattern acquisition and retention.

**Fig. 1:**
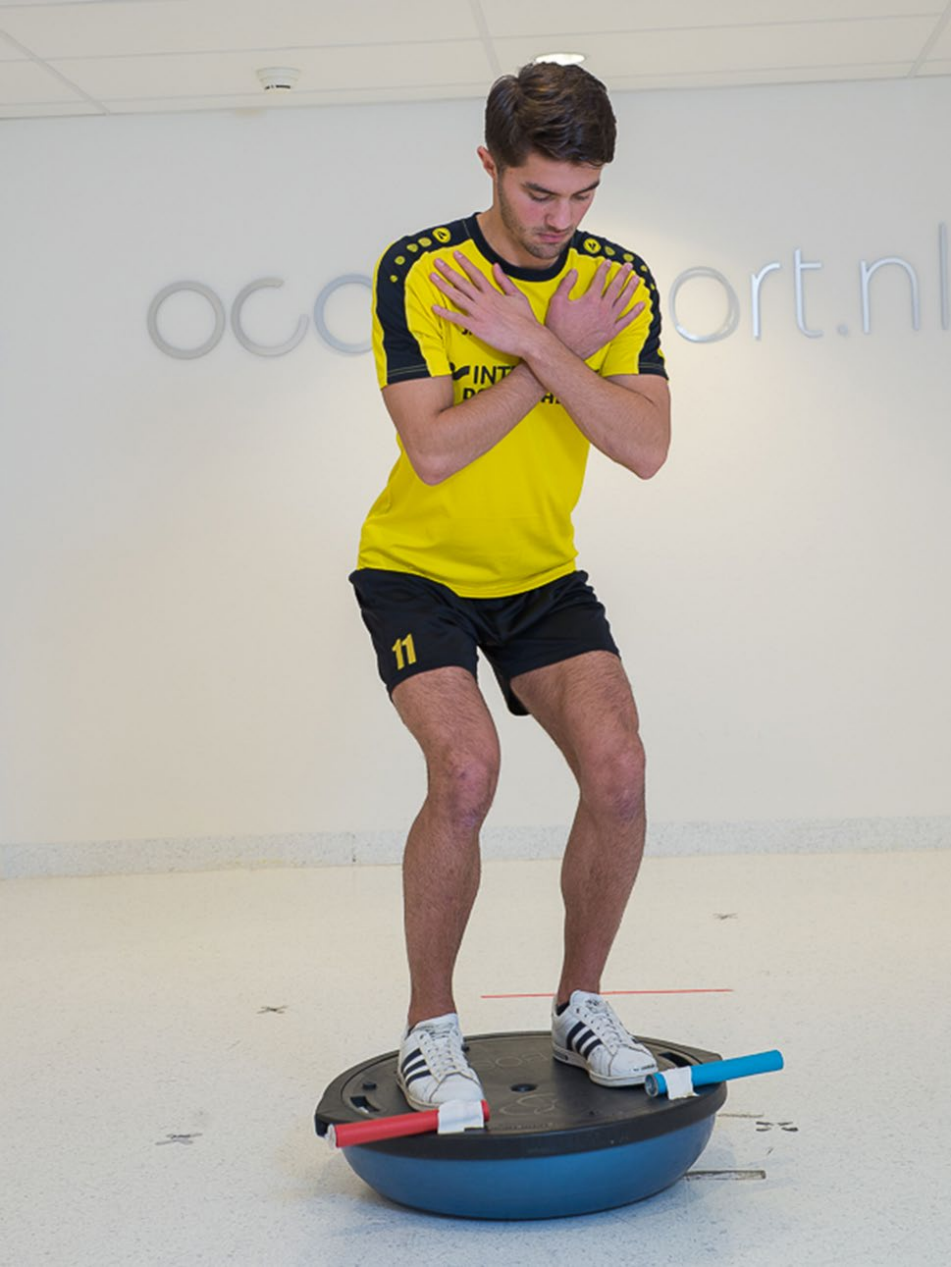
An external focus instruction to enhance postural stability “Try to keep the bars on the balance board as steady as possible”

**Fig. 2:**
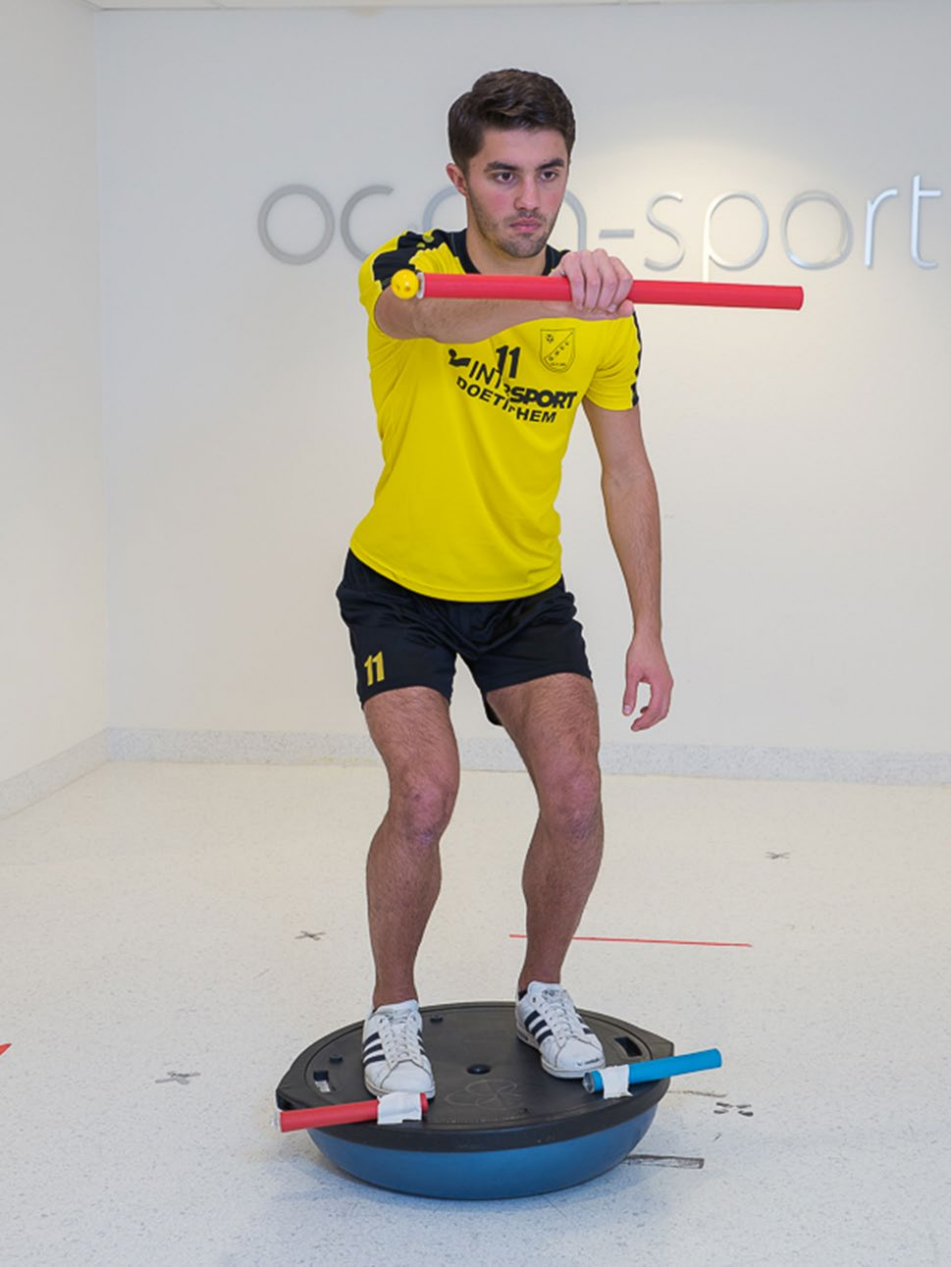
An external focus instruction to enhance postural stability “Keep the bar horizontal”

Examples of internal and external focus instructions (Figs. 1 and 2) are presented in Table 1 and illustrate how these can be easily adopted in daily clinical care.

**Table 1:** Comparison of instructions with internal focus and external focus

Goal: improve postural stability	Internal focus	External focus
Instructions	Try to keep your knee aligned over your second toe	Try to keep the bar horizontal
	Try to minimize movements of your feet	Try to minimize movement of the bars on the balance board
	Try to keep your balance by stabilizing your body	Try to keep your balance by stabilizing the platform

### Implicit Learning Enhances Movement Execution in Sports

The aim of implicit learning methods is to minimize the amount of declarative (explicit) knowledge about movement execution during learning. For this purpose, implicit learning can be induced by providing analogies rather than explicit instructions during the acquisition of motor skills [38]. Analogy, or metaphorical description of the action, connects with a visual image to help the patient ‘feel’ a movement.

Implicit learning reduces the reliance on the working memory and promotes more of an automatic process [38]. It is for this reason that it can be more effective in more complex tasks. Competitive sports can be psychologically demanding and decision-making accuracy deteriorates in athletes when they are under pressure and have to deal with increased task complexity [39]. The negative influences of pressure can be observed in several ways. Of particular interest in connection to learning is ‘re-investment’, when an athlete begins to direct attention to the skills and movements which should already be automatic, and do not need conscious control. This re-investment may cause the athlete to make sudden mistakes in technical actions, which are relatively simple and were performed, without error, a thousand times before [40]. Explicit learning can promote re-investment because the athlete reverts back to memory by a detailed, step-by-step instruction about movement execution, often in the form of verbal guidance. Under stress, an athlete may unwillingly start to follow this guidance and divide smooth and fluent execution into separate blocks that would be detrimental for expert performance. Additionally, such excessive attention to the technical details can draw working memory resources from other aspects of athletic performance [38].

One of the most interesting and widely unexplored aspects of implicit learning in rehabilitation is its connection with anticipation and decision making. This may be important in the late stages of rehabilitation when the patient is approaching the RTS phase. An athlete should be progressively exposed to physical, environmental, and psychological stressors that are comparable to those they will be exposed to in the sport they participate in. In light of secondary ACL injury prevention, training in this phase of the rehabilitation process should emphasize motor control factors such as anticipation, responses to perturbation, and visual-motor control within complex task environmental interactions. Implicit training using limited visual information improves athletes’ anticipatory skills [41]. In contrast, an explicit learning group that received specific kinematic information did not demonstrate any improvement in anticipatory skills [41], whereas improved anticipatory sensorimotor skills from implicit learning in rehabilitation may give the athlete an improved capability to anticipate the need for corrective motor actions and avoid a potential high injury-risk scenario.

#### Neural Correlates of Implicit Learning

Various studies pertaining to the neural mechanisms related to implicit motor learning have been conducted revealing a unique neural network associated with implicit relative to explicit learning [40, 41]. In a recent study, subjects performed a serial reaction-time task in which visual cues were presented in a predetermined order [42]. The subjects learned the order of sequence implicitly and explicitly. Increases in motor-evoked potential (MEP) latency without a change in MEP amplitude were found for implicit learning conditions, but not the explicit session. These authors suggest that the acquisition of implicit knowledge involves the reorganization of the corticomotor pathway [42]. While MEP latency has not been associated with ACLR, alterations in corticospinal excitability are more commonly reported, even after completion of rehabilitation. Thus, while implicit learning may not target the specific corticospinal mechanisms, it may provide a means to influence corticospinal function more generally after ACLR [43].

In a laparoscopic simulation task, novice participants were assigned to an explicit or an implicit motor learning condition [44]. To determine whether implicit and explicit training resulted in different levels of neural coactivation, EEG coherence analysis was performed. Movement accuracy was similar in both groups after practice. However, the implicit group had low conscious awareness of what was learned. The main advantage of low conscious awareness is that greater neural efficiency allows athletes to deploy resources to other aspects of performance [44]. This is particularly vital when athletes return to sport after ACLR as they must be able to maintain motor control of the injured joint whilst in the complex sport environment as ACLR has been associated with depressed sensorimotor neural efficiency in knee motor control [45]. Specific to neuromuscular training for ACL injury prevention, a recent report indicates engaging with implicit-based motor feedback during movement training can facilitate motor cortex efficiency for knee motor control, potentially providing a mechanism to improve not only motor coordination but address underlying nervous system dysfunction that remains with current standard therapy [46].

#### Clinical Examples

Examples using analogies and metaphors (implicit instructions) are provided in Table 2. A comparison is provided by presenting explicit, internal focus instructions commonly given during rehabilitation.

**Table 2:** Use of explicit and implicit instructions for exercises commonly done in rehabilitation

Task	Explicit instructions	Implicit instructions
Squat	Stand with your feet shoulder-width apart	Stance: Think about keeping a big ball between your knees
	Lower down so your thighs are as parallel to the floor as possible, with your knees over your ankles	Imagine you’re picking up a heavy box from the floor
		Imagine you’re going to sit down on a chair
Running	Bend your knees while landing	Imagine you run like a feather
		Land softly
		Try to make as little noise as possible
Vertical jump	Bend your knees before you jump Explosively extend hips, knees, and ankles, and propel off balls of feet to jump straight up Landing: bend knees during landing Keep your knees over your toes	Imagine you’re landing on eggs and you don’t want to crack them Push yourself off the floor as hard as possible Pretend you are a rocket that launches
Countermovement jump	Stand with your feet shoulder width apart Bend your knees before you jump Explosively extend hips, knees, and ankles, and pull your thighs towards your trunk Land with your knees bent	Imagine you’re jumping on hot coals and don’t want to burn your feet Push yourself off the floor as hard as possible Pretend you are a rocket that launches

### Differential Learning Supports Self-Organized Learning Process

Differential learning is based on the theory of dynamical systems [47]. When using differential learning in the practice of movement skills, the movement patterns themselves are intentionally varied during practice. This theoretical principle suggests that by having athletes perform a variety of movement patterns, a self-organized process of learning is initiated [47]. Through the process of experimentation with different movement patterns, target goals, and by learning alternative means of performing a task (rather than only practicing the supposedly ‘correct’ movement form), athletes learn an individualized motor solution that works best for themselves given the environmental context and constraints of their own bodies [48]. For example, during practice of a broad jump, the athlete performs multiple variations. The same variations are not repeated more than twice. Another method to ensure differential learning is, for example, engaging in environmental variations. This can ensure that motor patterns learned in the clinical setting can be transferred in a variety of circumstances and contexts. The concept is that practice should involve exposure to as many different combinations within a class (e.g., jumping, throwing, running) of skills as possible. The athlete must learn how to alter a particular movement strategy to achieve a particular outcome in different conditions. Although the scientific literature in this area is scarce, differential learning may have important clinical benefit [49].

#### Neural Correlates of Differential Learning

The nature of the neurophysiological processes underlying differential learning versus repetitive practice has been examined recently in badminton players [50]. The subjects performed badminton serves in differential learning and repetitive training schedules. EEG activity was recorded before and immediately after each 20-min exercise. Increased theta activity was obtained in contralateral parieto-occipital regions after differential learning. Further, increased posterior alpha activity was obtained in differential learning compared with repetitive training. The brain activation patterns indicate somatosensory working memory processes where attentional resources are allocated in the processing of somatosensory information in differential learning. Reinforcing a somatosensory memory trace might explain increased motor learning rates in differential learning. Finally, this memory trace is more stable against interference from internal and external disturbances that afford executively controlled processing such as attentional processes. The clinical relevance for athletes is that they would have attentional resources available that would allow for anticipation of potential high-risk situations, giving them an opportunity to avoid this situation or, if time is limited, pre-activate the neuromuscular system using feed-forward mechanisms.

Differential learning is not the standard in rehabilitation, which typically consists of one exercise for a predefined number of repetitions and sets, before moving on to the next exercise. However, in most sports it is quite rare to repeat the same movement for 3 sets of 10 repetitions before moving on to another movement, given that athletic activity requires rapid and variable movement performances that can be facilitated with differential learning approaches to therapy design. It is possible that neuroplasticity following ACL injury is in part due to therapy that does not engage differential learning. As Lepley et al. [43] showed, over the course of rehabilitation following injury, excitability of the motor cortex for quadriceps contractions decreased, which could in part be due to lack of differential exercise approaches that do not force the motor cortex to reintegrate the memory trace for quadriceps motor control before each repetition. While neurophysiological data across the stages of rehabilitation are lacking, with the investigation by Lepley et al. [43] being one of the only to quantify neuroplasticity longitudinally after the injury, the ability of differentially learning to modify sensorimotor neural processing may provide a means for therapists to target neural activity that standard therapy does not. Possible variations using differential learning for the practice of a jump task are presented in Table 3.

**Table 3:** Examples of how differential learning can be applied to practicing a double-legged jump task

Variations of the double-legged jump task	Change of environment	Change of athlete
Jump as far as you can. While you are jumping: Before jumping, 2–3 bunny hops, skipping both legs, skipping left leg, skipping right leg, high knees, left high knee, right high knee, butt-kicks both legs, butt-kicks left leg, butt-kicks right leg, zig-zag, shuffle to left, shuffle to right Make a full turn to left, to right before you jump While jumping, keep arms across the chest, behind back, raise left arm, raise right arm, circle both arms, circle left arm, circle right arm Move your head to left, to right Close left eye, close right eye While landing, one arm in front of and the other arm behind you Land with a very wide stance or with a very narrow stance Land on toes while landing	Exercises in dark Exercises on sand With shoes or without shoes In an environment with loud music or noise from audience in stadium In a virtual reality environment	Perform exercises with fatigue Perform exercises without fatigue Perform exercises with weighted vest

### Organization of Rehabilitation: Self-Controlled Learning and Contextual Interference

#### Self-Controlled Learning

**Fig. 3:**
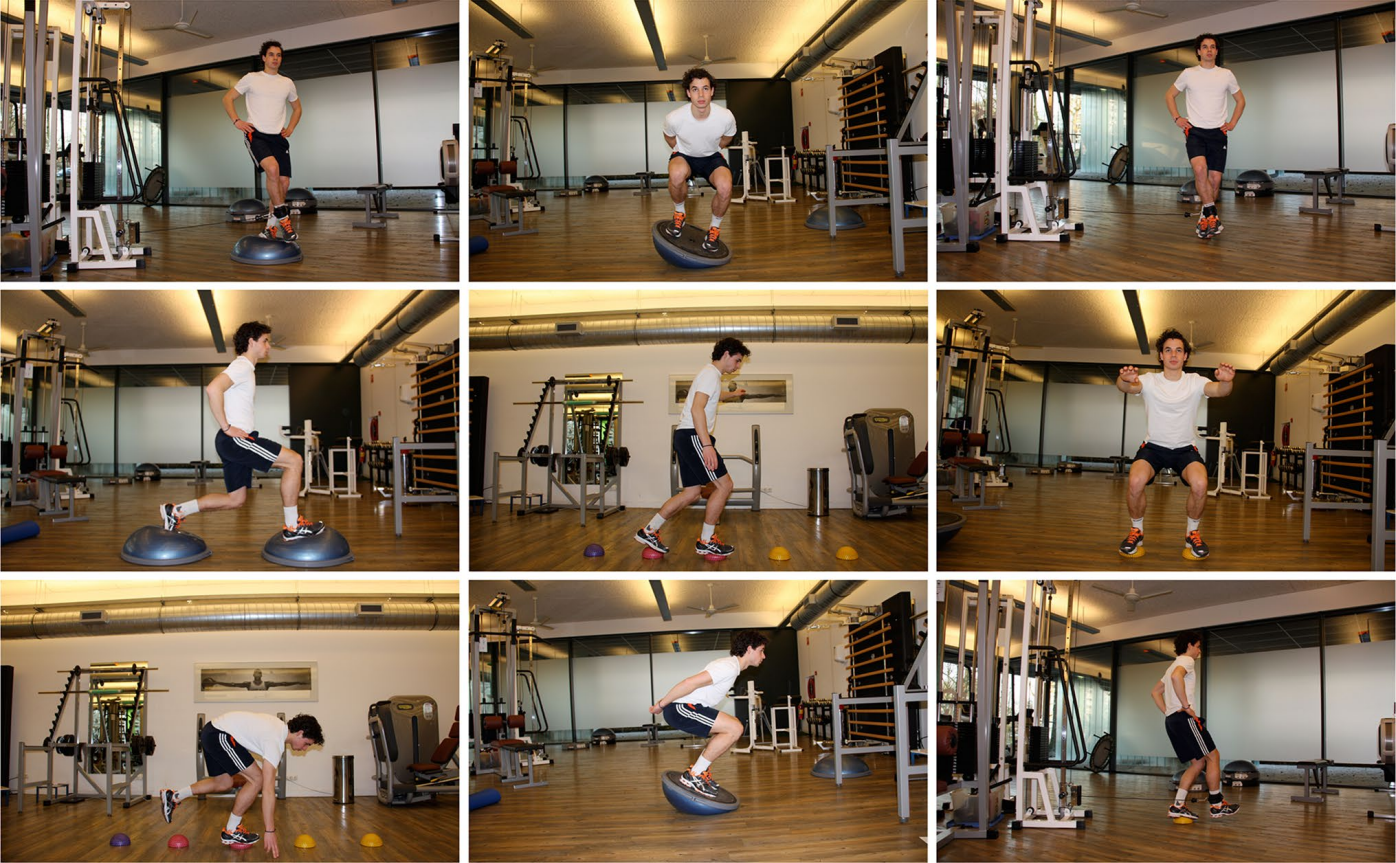
Self-controlled learning. The patient may choose, for example, three out of nine available exercises in the order they prefer

In most rehabilitation situations, clinicians determine the details of the training session. For example, they decide in which order tasks are practiced, the practice duration, and when or if instructions or demonstrations will be given. Thus, while clinicians generally control most aspects of practice, patients assume a relatively passive role. Self-controlled learning (e.g., giving the patients [some] choice to request feedback or choose an exercise) is a powerful tool in motor learning [29]. An example is provided (Fig. 3) of how self-controlled learning can be applied, using a poster depicting nine available exercises. For example, patients can choose three exercises for any given rehabilitation session.

**Fig. 4:**
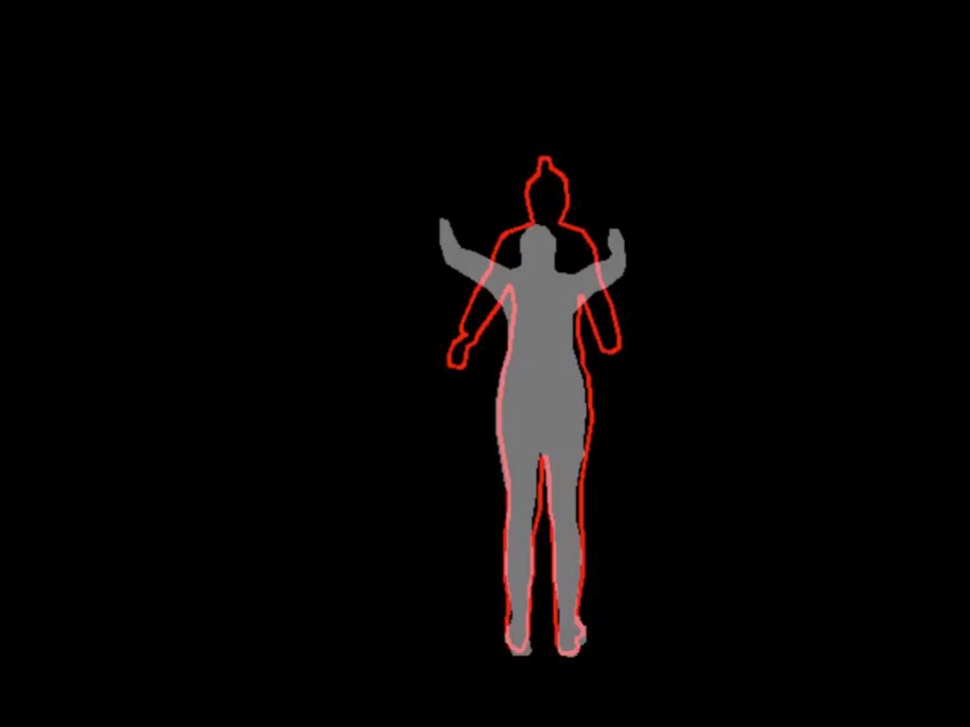
Video overlay of the model performing a drop vertical jump. Immediately after the drop vertical jump, the patient can view the overlap and try to increase their overlap with the model in the next jump

Clinicians can also encourage patients with positive feedback to enhance feelings of success to optimize motor learning. For example, to instruct athletes on the proper jump-landing technique, a recent study employed visual instruction in the form of an expert model performing a jump-landing task [51]. The athletes watched the contour of an expert on a television screen who performed an optimal drop vertical jump. Athletes were instructed to create as much overlap as possible with the expert model. In terms of self-control, they were allowed to request feedback when they wanted to see how closely they were replicating what the expert did (Fig. 4). Of note, the majority of athletes requested feedback after good trials. Athletes have a preference to receive positive feedback, which supports the motivational influences on motor learning by reinforcing good trials [52, 53]. Experiencing competence through feedback on good trials positively affects motor learning through motivational influences such as intrinsic motivation, interest, and enjoyment [54]. Self-controlled feedback schedules have the potential to help patients become more involved in their learning process [29, 55] by facilitating an active role during practice sessions that enhances motivation and increases effort and compliance [56–59]. This self-controlled feedback schedule is suggested to positively influence the motor learning process as it can be tailored to individual patients’ needs as opposed to depending on generic predetermined feedback schedules [55, 59, 60].

#### Neural Correlates of Self-Controlled Learning

The neuroplasticity associated with self-controlled learning was also apparent in a recent EEG study in which participants were assigned to either self-control or yoked groups and asked to practice a tossing task [61]. Self-control participants received augmented feedback at their discretion, whereas yoked participants were given feedback schedules matched to self-control counterparts. The subjects in the self-control group had better accuracy on the transfer of a newly acquired motor skill, which is an indication of connecting neuroplasticity with behavioral gains [61]. In another study, the role of autonomy in promoting performance on self-regulation tasks was examined in relation to the neural mechanism involved [62]. Task performance was positively related to increased event-related negativity (ERN) magnitude, which was positively related to performance [62]. The amplitude of the ERN is a sensitive measure of the intent and motivation of participants [62]. It has been demonstrated that motivation improves motor skill learning [63], which is highly relevant in ACL rehabilitation.

### Contextual Interference

Practice has a key role in the acquisition of motor skills during rehabilitation. Hence, the way the practice is scheduled influences the acquisition of motor skills. Three categories are used to describe variability and practice order: blocked, serial, and random. Traditionally, practice is scheduled in a blocked (constant) fashion. Blocked practice is when an athlete performs a single skill over and over, with repetition being the key. Variance in training is minimized or nonexistent. In serial practice, a certain prearranged series of tasks are repeated and practiced. Random practice involves practicing multiple skills in a random order. Variable practice involves performing variations of the task or completely different tasks throughout a treatment session. For transfer of skill to occur, a review of the literature has suggested that variable practice may be more effective [64].

Contextual interference in motor learning is defined as the interference in performance and learning that arises from practicing one task in the context of other tasks [64]. The amount of contextual interference may vary, between low in blocked practice and high in random practice. Practice under conditions of high contextual interference (i.e., with a random practice order) degrades performance during acquisition trials compared with low contextual interference conditions (i.e., with a blocked order, where practice is completed on one task before practice on another task is undertaken) [64]. Bjork proposed the concept of ‘desired difficulties’ that refers to practice conditions that engage the learner in an effortful learning process and have been shown to enhance retention and transfer [65].

For clinical application, skill level of a patient is a factor that may need to be considered in terms of amount of contextual interference provided. During blocked practice there is low interference or disruption in memory as a person practices multiple trials repeatedly. However, in random practice there is high interference because trials are interrupted by other tasks. Study results have shown that while higher contextual interference (random practice) may lead to poor(-er) performance, it frequently leads to better learning (as measured by retention and transfer tests) compared with blocked practice [66, 67]. This may occur because in random practice the skill must be reconstructed on each attempt, allowing an individual to practice a variety of strategies. Clinicians must decide how to best schedule practice to facilitate learning. As mentioned, the skill level of a patient is a factor that may need to be considered in terms of amount of contextual interference provided [66]. In general, lower level athletes benefit more from low contextual interference, whereas elite athletes do well with high levels of contextual interference [64, 68, 69].

#### Neural Correlates of Contextual Interference

The neural substrates of contextual interference during motor learning have been examined in a study with participants assigned to a blocked group or contextual interference group, learning a sequential task with the left hand [70]. Based on the fMRI data, the random group showed greater activity in sensorimotor and premotor regions compared with the blocked group. These areas are associated with motor preparation, sequencing, and response selection with greater activity in the random group potentially indicating more precise movement preparation and a requirement to reproduce the entire neural activity cascade to generate the motor performance, whereas the blocked group could simply reproduce the near final stages of neural processing to continently reproduce the motor performance.

Contextual interference may provide a unique method to address the visual–motor and sensorimotor specific neuroplasticity after ACL injury as described by Kapreli et al. [71] and Grooms et al. [45]. Both reports, despite one being in ACL deficiency [71] and the other in patients after ACLR [45], found increased reliance on visual input and cortical motor planning for the control of knee movement. The use of contextual interference methods may provide a means for clinicians to shift the relative level of sensory weighting (relative utilization of proprioceptive or visual input) for motor control. Contextual alterations could be as simple as slight variations to the environment (taking the athlete to the field or court) or changes in the sensory demands for the exercise.

## Motor Learning in ACL Injury Rehabilitation

In a frequently observed injury mechanism, the player is embedded in a situation where external factors such as possession of a ball and position of team mates and opponents are involved [72]. The attentional and environmental components of neuromuscular function are largely not addressed in current ACL rehabilitation programs. The authors feel that more emphasis should be given to the integration of sensory–visual–motor control factors during rehabilitation such as reaction time, information processing, focus of attention, visual–motor control, and complex-task–environmental interaction [16, 73]. This is particularly important in the late stages of rehabilitation [16].

Rehabilitation programs mainly focus on pre-planned motor skills in a predictable environment with a focus on postural alignment [21, 74]. Practicing these closed motor skills fails to comprehensively address the interaction between sensory cues and motor responses as they relate to specific sports activities of an athlete in task and environmental constraints on the field [75]. An athlete after ACL injury should be progressively exposed to physical, environmental, and psychological stressors to which they will be exposed, in the sport that they will be returning to, as part of a comprehensive and progressive RTS continuum [76].

## Summary

The purpose of this article is to present perspectives from motor learning to support neuroplasticity applied to clinical settings that have the potential to improve rehabilitation strategies and reduce the risk of second ACL injury. There are many variables to consider when structuring training programs for patients after ACLR, including the type, amount, intensity, and frequency of exercise and the importance of minimizing adverse reactions such as pain or swelling. From a motor learning perspective, people vary in their ability to learn new motor skills [77].

Clinicians should be cognizant that patients need to find an individualized motor solution that works best for them within their specific environmental context and the constraints of their own bodies [48]. In this manuscript, various principles of motor learning have been presented. However, not all of the described approaches of motor learning can be combined simultaneously within a single training session. The exact decision as to when and which approach is chosen requires good communication between the patient and the clinician.

## Future Directions

A strong link has been demonstrated between acquisition of motor skills and neuronal plasticity at cortical and subcortical levels in the central nervous system that evolves over time and engages different spatially distributed interconnected brain regions [78].

Evidence is emerging indicating the large cascade of neurophysiological alterations that occur after ACL injury. Although considered a unilateral injury, an ACL injury induces bilateral lower extremity dysfunction [12, 79], with sensory information deficits across the whole spectrum of the sensorimotor system, lending further support to the theory of a neurophysiological lesion [71, 80, 81].

Grooms et al. [45] posited that rehabilitation in patients after ACL injury should include sensory modifications to decrease the dependency of patients on visual information and in turn facilitate neuroplasticity. Another possibility is that patients have ineffective motor learning strategies and/or that motor learning to (re-) acquire motor skills is not sufficiently stimulated during traditional rehabilitation [73]. Such evidence could help to explain why patients do not always regain motor skills after ACL injury as the neuroplastic capacities may not be optimally challenged in current rehabilitation. In this manuscript, principles of motor learning have been presented to be used in rehabilitation after ACL injury.

Use of the principles of attentional focus seems to be very promising in clinical settings. In a systematic review, it was clearly established that using instructions with an external focus resulted in better motor performance and quality of movements (improvements were sustained over time) compared with an internal focus of attention [32]. Adopting an external focus results in greater knee flexion angles, increased center of mass displacement, lower peak vertical ground reaction force, and improved neuromuscular coordination, while maintaining or improving performance (e.g., jump height, jump distance) [32]. These findings are promising, representing an optimum of diminishing ACL injury risk (i.e., improved movement skills) without reduction in performance [32].

Although behavioral studies have clearly indicated that external focus instructions outperform internal focus instructions in learning motor skills, the mechanisms behind these effects are still an area that warrant further research. More specifically, more fundamental research is needed to determine what the underlying brain principles are. The literature is also scarce pertaining to the other principles of motor learning outlined in this manuscript. Our lack of understanding of the neurophysiological processes involved in application of external focus attention applies also to our understanding of the mechanisms involved in implicit and differential learning. Given the reorganization of the central nervous system that takes place after an ACL injury, we need to determine which principles of motor learning could enhance the neuroplastic processes that could translate to motor learning interventions with the goal of optimal function of the patient.

## Conclusions

The current evidence suggests that an increased risk of a second ACL injury is highly related to asymmetrical joint loading. Current rehabilitation programs appear not to be effective in targeting these aberrant movement patterns. Several novel motor learning principles have been presented in this article. We have provided a continuum of recently developed neuromuscular training programs using novel training methods for high-risk populations, now tailored to target biomechanical and neuromuscular risk factors as identified in patients after ACLR. Future research should focus on which, if any, combinations of the presented novel motor learning principles yield better clinical outcomes. Motor learning should be applied to support neuroplasticity after ACL injury. Because every person (and brain) is different, the optimal solution may require motor learning principles individually tailored to the injured athletes.
